# How the Psychology of Education Contributes to Research With a Social Impact on the Education of Students With Special Needs: The Case of Successful Educational Actions

**DOI:** 10.3389/fpsyg.2020.00439

**Published:** 2020-03-13

**Authors:** Elena Duque, Regina Gairal, Silvia Molina, Esther Roca

**Affiliations:** ^1^Department of Theory and History of Education, University of Barcelona, Barcelona, Spain; ^2^Department of Pedagogy, Universitat Rovira i Virgili, Tarragona, Spain; ^3^Departament of Comparative Education and Education History, University of Valencia, Valencia, Spain

**Keywords:** social impact, psychology of education, special educational needs, interactive groups, dialogic literary gatherings

## Abstract

One current challenge in the psychology of education is identifying the teaching strategies and learning contexts that best contribute to the learning of all students, especially those whose individual characteristics make their learning process more difficult, as is the case for students with special needs. One main theory in the psychology of education is the sociocultural approach to learning, which highlights the key role of interaction in children’s learning. In the case of students with disabilities, this interactive understanding of learning is aligned with a social model of disability, which looks beyond individual students’ limitations or potentialities and focuses on contextual aspects that can enhance their learning experience and results. In recent years, the interactive view of learning based on this theory has led to the development of educational actions, such as interactive groups and dialogic literary gatherings, that have improved the learning results of diverse children, including those with disabilities. The aim of this paper is to analyze the social impact achieved by a line of research that has explored the benefits of such successful educational actions for the education of students with special needs. National and European research projects based on the communicative methodology of research have been conducted. This methodology entails drawing on egalitarian dialogue with the end-users of research – including teachers, students with and without disabilities, students’ relatives and other community members – to allow an intersubjective creation of knowledge that enables a deeper and more accurate understanding of the studied reality and its transformative potential. This line of research first allowed the identification of the benefits of interactive learning environments for students with disabilities educated in mainstream schools; later, it allowed the spreading of these actions to a greater number of mainstream schools; and more recently, it made it possible to transfer these actions to special schools and use these actions to create shared learning spaces between mainstream and special schools. The improvement of the educational opportunities for a greater number and greater diversity of students with special needs evidences the social impact of research based on key contributions of the psychology of education.

## Introduction

Access to mainstream, inclusive and quality education for children with disabilities has not yet been fully achieved. Children with disabilities are still being educated in special schools in most countries, with varying percentages depending on the country, and therefore these schools attend diverse special needs (World Health Organization, 2011). In addition, students with disabilities and special needs tend to leave school without adequate qualifications (European Agency for Special Needs and Inclusive Education, 2017). Therefore, the appropriate inclusion of children with disabilities into the general education system is part of the European Disability Strategy 2010–2020 (European Commission, 2010). In this context, one current challenge of the psychology of education is to identify the teaching strategies and learning contexts that best contribute to the education of students with special needs. In this endeavor, research in the psychology of education is focused on the strategies, actions and practices that enhance the learning of these students, taking into account their individual characteristics, however, importantly, research is also focused on the strategies, actions and programs that benefit the learning of all students, including those whose individual characteristics make the learning process more difficult, so that shared learning environments that promote successful learning for all can be created.

Instrumental learning, especially in regards to difficulties in reading and literacy, is one of the main concerns of research on the psychology of education (Lloyd et al., 2009; Alanazi, 2017; Alenizi, 2019; Auphan et al., 2019; Hughes et al., 2018). Numerous programs for improving reading and/or reading difficulty prevention have emerged from research on reading and literacy from the perspective of the psychology of education, and their impact on improving children’s learning has been analyzed (Vellutino and Scanlon, 2002; Papadopoulos et al., 2004; Hatcher et al., 2006). There are also specific studies about reading and literacy programs and their success with students with special needs (Holliman and Hurry, 2013) and/or with students at risk for reading disabilities (Lovett et al., 2017). Strategies to promote the learning of mathematics in children with special educational needs and disabilities have also been studied (Pitchford et al., 2018), and programs based on these strategies have been developed (Montague et al., 2014).

Research has also explored the association between learning difficulties and behavior problems (Roberts et al., 2019), showing that lower academic achievement is a risk factor for developing behavior difficulties among students with special educational needs and disabilities (Oldfield et al., 2017). The study of the learning context and the school environment, which facilitates or hinders learning, has shown that the expectations from teachers and their attitudes toward children with special needs are some of the most influential elements (Anderson et al., 2014; Wilson et al., 2016; Bowles et al., 2018). Research has also found that teachers can have an important influence on the social acceptance of peers with special needs (Schwab et al., 2016), which is important because the social exclusion of children can affect their learning difficulties and behavior problems (Krull et al., 2018). The efficacy of peer network interventions for improving the social connections of students with severe disabilities has been highlighted (Asmus et al., 2017), and programs and educational actions based on peer interaction, such as cooperative learning (Velázquez Callado, 2012), have been developed to improve the school climate. Importantly, there are effective programs for improving peer acceptance and a positive coexistence related to curricular learning (Law et al., 2017; Vuorinen et al., 2019), which is a key issue in facilitating inclusive education.

This body of research on effective actions and programs to enhance the learning and inclusion of students with disabilities and special needs shows the capacity that research in the psychology of education has for improving the education of these students. It also shows the importance that the learning context has, regarding both instruction and social relations, on the academic and social performance of students with special needs. This resonates with the social model of disability, an approach that has been claimed, from the perspective of human rights, to shift the focus from non-disabled centrism and to transcend the traditional and individualistic perspective of disabilities to focus on the improvement of educational experiences for these students (Chun Sik Min, 2010; Park, 2015). This perspective assumes not only that children with disabilities should be included in mainstream education but also that inclusive education can be more effective (Lindsay, 2007). This interactive understanding of learning allows seeing beyond individual students’ limitations or potentialities and focusing on contextual aspects that can enhance their learning experience and results (Goodley, 2001; Haegele and Hodge, 2016).

The classical psychology of education already emphasized the importance of the social context for children’s learning. In particular, the sociocultural approach of learning developed by Vygotsky and Bruner highlighted the key role of interaction in children’s learning and development. Both authors agreed that what a child learns has been shared with other persons first, emphasizing the social construction of knowledge. While Vygotsky (1980) stated that in children’s development, higher psychological functions appear first on the interpsychological level and then on the intrapsychological level, Bruner (1996) refers to a social moment where there is interaction and then an individual moment when interiorization occurs.

Bruner evolved from a more cognitivist perspective of learning centered on individuals’ information processing (Bruner, 1973) to a more sociocultural and interactive perspective (Bruner, 1996) within the framework of which he conceptualized the idea of “scaffolding,” which enables novice learning in interaction with an expert, and “subcommunities of mutual learners,” where “learners help each other learn” and “scaffold for each other” (Bruner, 1996, p. 21). For Bruner, “It is principally through interacting with others that children find out what the culture is about and how it conceives of the world” (1996, p. 20); therefore, learning occurs through interaction within a community.

Vygotsky stated that learning precedes development, not the other way around, and he conceptualized the zone of proximal development (ZPD), which represents the opportunity that learning interactions with adults and more capable peers have to advance children’s development (Vygotsky, 1980); beyond the actual level of development, the ZPD emphasizes the importance of interactions with others to solve problems and learn. He emphasized that this interaction is especially important for children with disabilities: “Precisely because retarded children, when left to themselves, will never achieve well elaborated forms of abstract thought, the school should make every effort to push them in that direction and to develop in them what is intrinsically lacking in their own development” (Vygotsky, 1980, p. 89). In this regard, he warned of the risks of working with children with disabilities from a perspective centered on biological processes and basic dysfunctions instead of working with higher psychological functions (Vygotsky, 2018). Vygotsky’s focus on interaction provides new opportunities for learning and development for children with special needs to develop these higher psychological processes.

The sociocultural approach of learning developed by Vygotsky and Bruner has continued inspiring theory and research in the psychology of education to today. According to Dainez and Smolka (2014), Vygotsky’s concept of compensation in relation to children with disabilities implies a social formation of mind and therefore the social responsibility of organizing an appropriate educational environment for these children. Vygotsky’s approach has been taken into account in studies about how peer mediation increases learning, especially when peers have different cognitive levels (Tzuriel and Shamir, 2007), and research on children with disabilities, for instance, cerebral palsy, has been conducted based on Vygotsky’s contributions and showed improvements in these children’s spatial abilities, social interaction, autonomy, and participation in class activities (Akhutina et al., 2003; Heidrich and Bassani, 2012).

In recent years, the interactive view of learning has led to the development of educational actions that have improved the learning results of diverse children, including those with disabilities. INCLUD-ED (Flecha, 2006-2011) was an integrated project funded by the European Union under its 6th Framework Programme with the main objective of achieving both academic success and social cohesion for all children and communities in Europe, regardless of their socioeconomic status and/or ethnic background. INCLUD-ED identified successful educational actions (SEAs), that is, actions that can improve school success and contribute to social cohesion in every context where they are implemented (Flecha, 2015). Some of the SEAs that have demonstrated improvements in reading, mathematics and peer relationships include interactive groups (IG) and dialogic literary gatherings (DLG). IG (Valls and Kyriakides, 2013) consists of organizing classrooms in small heterogeneous groups that work on instrumental learning activities drawing on mutual support and dynamized by adult volunteers from the community; DLG (Soler, 2015; Lopez de Aguileta, 2019) consists of reading and discussing classical works of literature based on the principles of dialogic learning, reaching deeper understanding of the texts as a result of sharing the participants’ interpretations and meanings. In both actions, learning interactions, as the main tool to promote learning, are facilitated among diverse persons in accordance with the contributions of the sociocultural theory of learning. In this regard, previous research has identified that Vygotsky’s and Bruner’s contributions are at the basis of these SEAs (Elboj and Niemelä, 2010; Garcia et al., 2010).

## Materials and Methods

The objective of this paper is to analyze the social impact achieved by a line of research that has explored the benefits of SEAs on the improvement of the education of students with special needs. For this purpose, the following data collection methods were used. First, existing data from case studies conducted within the four projects that compose this line of research have been analyzed to identify the impact of SEAs on students with special needs. These projects studied the benefits of SEAs for diverse students at different specific levels (i.e., school and classroom organization, community participation, interactions). In this paper, we aim to go beyond these specific aspects to understand in a more integrated and comprehensive manner how these different levels contribute to the impact that SEAs have, specifically on students with special needs. Second, new data were collected through in-depth interviews with teachers involved in the implementation of these actions in their schools as a consequence of this line of research. These interviews allowed the analysis of the subsequent impacts achieved as a result of conducting research on this topic from the perspective of the agents involved in the implementation of the results of this line of research.

All participants (teachers, volunteers, families, and children) agreed to provide researchers access to relevant data for the purpose of the study. Prior to data collection, they were informed of the nature of the research, and written informed consent was obtained. In the case of minors, informed consent was obtained from their parents or guardians. All participants were informed that their participation was anonymous and voluntary and that data would be treated confidentially and used solely for research purposes. Ethical requirements were addressed following the Ethics Review Procedure established by the European Commission (2013) for EU research, the Data Protection Directive 95/46/EC and the Charter of Fundamental Rights of the European Union (2000/C 364/01). The study was fully approved by the Ethics Board of the Community of Researchers on Excellence for All (CREA)^1^.

### Case Studies

The line of research that we analyze in terms of social impact is composed of four national and European research projects in which the authors have participated in the last 15 years. In these projects, a total of 36 case studies were conducted. Of these cases, 10 included data on the participation of students with special needs in SEAs (see Table 1), and these were analyzed for the purposes of this paper. These cases fulfilled two criteria: (1) the schools were implementing SEAs and (2) students with special needs participated in SEAs with their classmates. Overall, 60 data collection techniques were used in the 10 case studies. These included 36 interviews, 14 with class teachers (3 of them were also special education teachers), 4 with special education teachers, 3 with volunteers, 8 with students, and 7 with students’ relatives; 13 focus groups, 5 with teachers, 8 with students, and 1 with students’ relatives; and 10 observations, 9 in classrooms and 1 in a teachers’ meeting (see more details in Table 1).

**TABLE 1:** Summary of the data collection instruments and participants in the project case studies.

Case studies	Instruments	Projects and timing
5 case studies of students with special needs participating in interactive groups with typically developing peers in primary education. Special needs included cerebral palsy, global developmental delay, visual impairment, and dyslexia (Catalonia and the Basque Country, Spain).	– Interviews with class teachers (3), special education teachers (3), volunteers (3) – Focus groups with teachers (2), students (5) – Structured observations (5)	*Interactive groups: a practice of learning communities for the inclusion of students with disabilities.* Agency for Management of University and Research Grants of the Catalan Government. (2003-2007)
1 case study of 1 primary school implementing successful educational actions, with students with and without special needs, including cerebral palsy, sensory impairments, brain injury, developmental disharmony, eating disorders, depression, and ADHD (Catalonia, Spain).	– Interviews with students (5), students’ relatives (5), class teachers (2), special education teachers (1) – Focus group with teachers (1) – Observations in classrooms (4), teachers’ meetings (1)	*INCLUD-ED. Strategies for inclusion and social cohesion in Europe from education* European Commission, 6th Framework Programme. (2006-2011)
3 case studies of 1 primary school and 2 secondary schools implementing successful educational actions, with students with and without special needs (Castilla-La Mancha, Basque Country and Andalusia, Spain).	– Interviews with teachers (6), students (3), students’ relatives (2) – Focus groups with teachers (1), students (1), relatives (1).	*MIXTRIN. Ways of grouping students together and how this is related to success at school: Mixture, Streaming and Inclusion*. R + D Plan. Spanish Ministry of Science. (2008-2011)
1 exploratory case study of a special school implementing successful educational actions for 2 years with students in primary and secondary education with disabilities including intellectual disability, autism spectrum disorder, and cerebral palsy (Valencian Community, Spain).	– Interviews with teachers (3) – Focus groups with teachers (1), students (2)	INTER-ACT. I*nteractive learning environments for the inclusion of students with and without disabilities: improving learning, development and relationships*. R + D Plan. Spanish Ministry of Science. (2018-2020)

The different projects focused on different aspects of the SEAs and therefore entailed different layers of analysis throughout this line of research, which has allowed a comprehensive view of the benefits of SEAs for diverse students and specifically for students with special needs.

The doctoral project funded by the Catalan Government (Molina, 2003-2007) was the first research to specifically focus on the inclusion of students with special needs in SEAs, and particularly analyzed the type of classroom interactions that facilitate students’ inclusion when classrooms are organized in IG. The project’s main objective was to analyze the influence that students’ participation in IG has on their educational inclusion. The main categories of analysis were *peer interactions* and *community participation* as components of IG and *learning*, *participation and social inclusion* as components of educational inclusion.

INCLUD-ED (Flecha, 2006-2011) aimed to identify educational actions that contributed to overcoming segregation and promoted the inclusion of all students in schools across Europe, with a special focus on vulnerable groups of students. INCLUD-ED clarified the distinction between mixture, streaming and inclusion (INCLUD-ED Consortium, 2009) as different ways of organizing student diversity and human resources with different consequences on students; distinguished different forms of family and community participation; identified educative, decisive, and evaluative forms of participation as those that had more impact on students’ success; and identified successful educational actions. The contribution of this project to this line of research was an analysis of SEAs at the level of school organization, resource management and community engagement. The main objective of the case studies within this project was to analyze components from educational practices that decrease the rates of school failure and those of the practices that increase them. The main categories of analysis were *inclusive practices* and *community participation*.

MIXSTRIN (Valls, 2008-2011), as a continuation of the INCLUD-ED research in the Spanish context, deepened the analysis of the different forms and consequences of mixture, streaming and inclusion from a mixed methods approach. Thus, this project focused on analyzing SEAs at the level of classroom organization. The main objective of the case studies was to identify how different ways of grouping students are related to students’ learning results. The main categories of analysis were practices of *mixture, streaming*, and *inclusion*.

Finally, INTER-ACT (Garcia-Carrion, 2018-2020) analyzes how SEAs are being implemented with students with disabilities in both mainstream schools and special schools, with the aim of transferring these actions and their benefits to new schools. The project’s focus of analysis is the interactions that occur in IG and DLG in both types of schools. The main objective of the case study conducted was to analyze in depth successful cases of schools implementing IG and DLG with students with disabilities to identify the best conditions for increasing the impact on the improvement of learning, development and relationships. The main categories of analysis were *characteristics of the interactive learning environment* and *improvements achieved.*

Within the different research projects, using the case study as a methodological approach has allowed understanding the reality of the object of study in context. Following Stake (2006), case studies were selected based on what information they could provide about the issue explored, in this case, the increase in the educational quality provided to students in SEAs, especially to those with special needs. In this regard, case studies were instrumental in providing insight into this issue. As a sum of individual research projects, the line of research presented here constitutes multicase research (Stake, 2006), where cases share similarities – e.g., data collection techniques, the population object of study and purpose – and allow understanding from the singularity of each case of the broader phenomenon that all of them are part of.

### In-Depth Interviews

Five interviews were conducted with teachers who fulfilled two criteria: (1) they were implementing SEAs with their students, including students with special needs, and (2) they had started to implement these actions as a consequence of the research line on SEAs and special needs, that is, after becoming aware of the evidence obtained on the benefits of SEAs for these students. Two of the interviewees were teachers at one school where one of the case studies was conducted while the other interviews were not related to the case studies. The interviews were conducted by one of the researchers at the end of the 2018–2019 school year, and at that time, the participants had been implementing SEAs for a period of 4–6 years (see Table 2). The interviews lasted between 20 and 55 min and were conducted at times and in places that were convenient for the participants. We introduced the interviews as follows: “In the last 15 years, a line of research has been conducted on the educational inclusion of students with special needs through SEAs. We are interested in gathering information on the social impact of this line of research.” Sample questions were as follows: “Can you identify some of those impacts (e.g., improvements in the learning of students with special needs or improvements in the schools’ approach to responding to students’ diversity)?”; “How has the line of research led/contributed to such impacts?”; “Have these impacts been transferred to different contexts or students with different characteristics?”; and “Have the impacts been sustained over time?” All interviews were audio-recorded and transcribed verbatim for subsequent analysis.

**TABLE 2:** Profiles of the participants in the in-depth interviews.

Person^a^	Profile	Topic of the interview
Sandra	Principal of a mainstream school. 6 years implementing SEAs	Impact of the research on SEAs and special needs on students with special needs in mainstream schools and on the school’s approach to educating special needs students.
Irene	Principal of a mainstream school. 6 years implementing SEAs	
Carmen	Teacher of a mainstream school. 6 years implementing SEAs	
Marta	Principal of a special school. 4 years implementing SEAs	Impact of the research on SEAs and students with special needs in special schools and on the school’s approach to educating special needs students.
Ana	Teacher of a special school. 4 years implementing SEAs	

*^a^Real names have been changed to pseudonyms.*

### Communicative Methodology

This line of research has been conducted based on the communicative methodology (Gómez et al., 2011). The data collection and analysis of the social impact achieved has also been conducted based on this methodology. The communicative methodology entails drawing on egalitarian dialogue with the end-users of the research – including teachers, students with and without disabilities, students’ relatives and other community members – to allow an intersubjective creation of knowledge that enables a deeper and more accurate understanding of the studied reality and its transformative potential (Gómez et al., 2012), therefore enabling greater social impact. Different studies have demonstrated the suitability of this methodology when researching vulnerable groups (Puigvert et al., 2012; Gómez et al., 2019), as well as the social impact that this methodology produces.

Following the communicative methodology, in this line of research, data collection techniques were aimed not only at gathering the individuals’ experiences and perceptions but also to discussing these experiences and perceptions with them in light of previous scientific knowledge on the issue and with the purpose of identifying both the exclusionary and transformative components of the reality studied. While exclusionary components refer to the barriers encountered by certain persons or collectives, for instance, educational barriers encountered by persons with disabilities, transformative components are those elements that contribute to overcoming these barriers, for instance, certain types of classroom organization or learning interactions. The objective of the dialogues held with end-users and other stakeholders in the research process is to agree upon these exclusionary and transformative components, which strengthens the validity of the research results and its potential social impact.

### Data Analysis

For this paper, the different case studies have been analyzed together to understand in an integrated manner how the different layers analyzed previously (school and classroom organization, community participation, interactions) contribute to the social impact of the implementation of SEAs with students with special needs. For this purpose, the existing data of the case studies were analyzed with a new set of categories that was created to examine this social impact. Taking into account that the main challenges in the education of children with special needs are their limited participation in normalized learning environments (World Health Organization, 2011), their lower educational levels achieved (European Agency for Special Needs and Inclusive Education, 2017) and their higher risk of being socially marginalized and bullied (UNESCO, 2017), the improvements in these domains constitute the social impact of the educational intervention aimed at students with special needs; therefore, the following were the basis for creating the categories for the analysis of the social impact of SEAs:

(1) Impact on students’ participation: characteristics of the successful educational actions that enable the participation of students with special needs.
(2) Impact on the cognitive dimension: improvements achieved in instrumental learning and cognitive development.
(3) Impact on the socioemotional dimension: improvements achieved in social cohesion and emotional/affective development.

The newly conducted interviews on the social impact of the line of research were analyzed with categories that take into account the social impact criteria identified by IMPACT-EV (European Commission FP7, 2014-2017) and used in SIOR (Flecha et al., 2015) regarding improvements, sustainability and transference. The fourth category emerged inductively from the data:

(1) Impact on students with special needs: improvements and sustainability,
(2) Impact on schools: improvements and sustainability,
(3) Transference to new contexts, and
(4) Factors supporting social impact.

The themes present in the transcripts were coded by the researchers on a line-by-line basis. A deductive, flexible approach was used for the coding to identify subthemes within the categories. Categories of analysis were applied to the transcripts by two independent coders to enhance the validity of the results. Consensus for the coding was achieved through discussion.

## Results

In the following, the social impact of the line of research is presented, which includes evidence on the benefits of SEAs for students with special needs and how such SEAs led to a new social impact on different schools, students, teachers and contexts. Three types of social impact are presented: (1) impact on students with special needs and their opportunities to participate, learn and have positive relationships in SEAs; (2) impact on schools’ and teachers’ approaches to meeting students’ special needs; and (3) the replicability of SEAs to new types of educational contexts and student populations. The factors that have enabled the achievement of these impacts are also reported.

### Social Impact 1: Enhanced Participation, Learning Opportunities, and Group Cohesion for Students With Special Needs in Successful Educational Actions

#### The Social Impact of SEAs

Previous analyses of the case studies showed that SEAs entail a more efficient organization of classrooms and schools, allowing a more inclusive education for a diverse student body, including students with special needs, who can benefit from enhanced access to the content of the general curriculum in a shared learning environment (Christou and Molina, 2009; Molina and Ríos, 2010). A key feature of the inclusive learning environments promoted in the SEAs is the diverse interactions promoted around learning among, on the one hand, students, as they are organized in heterogeneous groups and, on the other hand, relatives and other members of the community, who are welcome to participate in the students’ learning activities. These interactions are key components of the SEAs that have created new learning opportunities for students with special needs in mainstream schools and, more recently, in special schools (García-Carrión et al., 2016, 2018).

The analysis of the social impact of SEAs on students with special needs shows positive impacts in terms of the participation, learning and social inclusion of these students. Regarding student participation, the supportive peer interactions promoted within the SEAs and the participation of volunteers from the community, who ensured that these supportive interactions were implemented and provided assistance themselves when necessary, facilitated normalized and active participation in learning activities and natural support within the student group, which progressively made specific, individualized support less necessary (Molina, 2003-2007). For this to occur, the case studies showed the importance of the activities that students worked on in the IG being the same for all of them and of students with special needs not being given different activities in any case. The same occurred with DLG: all students participating in the gathering read the same book. The analysis showed that this was important because both IG and DLG work based on interactions and, if one student was given an activity or a book that was different than that of the other students, interaction of this student with the group would be easily broken. In some cases, adaptations were made regarding the way students accessed the material, interacted with it or produced an output or regarding the level of complexity required. However, the learning content was always the same to allow the maximum benefit from interaction and the highest possible level of attainment. Across the case studies, teachers reported that supportive interactions within heterogeneous groups in successful educational actions had been more effective than differentiated individual attention separated from the class, even in the cases when additional human resources were used. Therefore, SEAs have prevented reducing learning opportunities related to the segregation and individualization of educational measures often aimed at these students (Valls, 2008-2011; Flecha, 2015).

In terms of learning and cognitive development, the possibility of asking questions when necessary and constantly seeing and listening to peers working on the activity and talking about it helped students with special needs stay connected to the activity, understand it and do it (Molina, 2003-2007; Valls, 2008-2011). Learning progress was more evident in instrumental learning subjects (literacy, math), which are prioritized in IG and DLG. Specifically, due to the interactive and dialogical nature of both IG and DLG, communicative ability is one learning and development area in which students show a clear improvement. In this regard, for these children, DLG have meant the opportunity to broaden their vocabulary and gain a better understanding of the language structure, as they are able to listen more proficient children and adults, who also model language and help the students with special needs express themselves better (Garcia-Carrion, 2018-2020).

Regarding the social impact of SEAs on the social inclusion of students with special needs and group cohesion, it was observed that the participation of these students in regular class activities that IG and DLG facilitated contributed to considering these students as “one of the class” and not a “part-time student” who only shares part of their time and activities with their classmates. Beyond participation, SEAs gave students opportunities to interact with their peers and therefore to come to know better each other, ultimately building new friendships. Peer support and friendship that were learned in IG and DLG often extended beyond the class and beyond the context of school, creating new opportunities for both cognitive and social development, for instance, when students with special needs had the opportunity to share their doubts with their classmates when doing homework via telephone or social networks or to meet them at birthday parties (Valls, 2008-2011; Garcia-Carrion, 2018-2020).

#### Extending Improvements to More Schools and Students

The case studies showed ways in which the education of students with special needs improved in SEAs, as well as key components of these actions that explained the results. Both findings were crucial to extend these actions and their benefits to more children with special needs and thus for the social impact of the research.

The first time that primary schools were transformed into learning communities and implemented SEAs was in 1995 in Spain. There were five schools at that time. Ten years later, in 2005, there were 22 schools. After 10 more years, in 2015, the number reached 120 schools in different countries (Flecha, 2015). Today, 225 schools in Spain^2^, 49 schools in other European countries^3^, and 411 schools in Latin America^4^, each with diverse populations, have become learning communities through the application of successful educational actions. These data show that the INCLUD-ED project (2006–2011) was a turning point in the spread of SEAs in schools. The spread of the project also meant that these actions could reach more diverse students with special needs. The applicability of the SEAs with these students was usually a topic of debate among the teachers that incorporated these actions in their schools. When the knowledge of the evidence provided by the line of research reached the new schools, both teachers and the rest of community became more confident when including students with disabilities in IG, DLG, and other shared learning activities in the school. The different teachers interviewed explained that the implementation of SEAs in their schools has increased over time and so has the participation of students with special needs, which reaches 100% in some cases. As one teacher explained, the participation in SEAs prevents the need for individual support outside the classroom: “Out of all the classes, there is not any child that gets out of the classroom [to receive individual support] when they work on SEAs” (Sandra). The implementation of SEAs with students with special needs – as well as with the general population of students – has not only been sustained but has increased, as, for teachers, it is an efficient way to respond to these – and other – students’ needs:

In the school, almost all students with special needs participate in SEAs (.) From my experience I can tell you that I used to do an SEA session per week, then I did two, this year I have done three, and now I cannot imagine less than three, every time I need to do it more and more. (Carmen)

The benefits observed by research in the case studies then started to spread to more children in other schools. Two examples can illustrate these improvements. First, the case of a child with a severe neurological deficit, for whom participating in SEAs made it possible to transform the expectations that were imposed on his learning possibilities:

The neurologist said that he could not learn almost anything. literacy and all the learning, they saw it as impossible. but he has learnt to read (…) if we hadn’t known about it, that evidence about interactions… Last year we did 6 sessions [of IG] per week, plus DLG, we did as much as we could, and it is amazing what he has learnt. (Sandra)

Research has already shown that being able to participate and learn in IG and DLG changes the self-concept and learning expectations of the children with special needs as well as the concept and expectations their peers and adults have of them. In these interactive learning environments, students were often able to solve tasks that they could not solve alone or read books that could not read alone, going beyond teachers’ and families’ expectations for their learning (Flecha, 2015). When SEAs reached new schools and students, these higher expectations, which create new learning opportunities, were created there too.

The second example is of a child with Down syndrome who could benefit from a more normalized learning environment where he could make progress in both learning and group belonging; this shows how the benefits that research had identified in group cohesion were replicated in other cases like this:

We had another child with a disability who was very isolated from the group, he did not have an emotional bond either, and the attention he received was too individualized; with the special education teacher, the speech therapist, he did not feel he belonged to the group. And when we started implementing SEAs, work in IG, DLG, the group changed very much, (…) and the child, who had Down syndrome, started to belong to the group: worked on the same activities as the others, and the others counted on that child. It was a huge change. (…) We achieved a lot of things. (Carmen)

Both teachers, Sandra and Carmen, clearly attributed the improvements observed to the students’ participation in IG and DLG. In some cases, looking for the participation of these children in SEAs has made teachers look for adaptations that enabled their participation. This was the case for a child in Irene’s school. He had not developed oral language, which made it difficult to participate in DLG, but the teachers adapted the book to pictograms and facilitated him in using a tablet with the pictograms and synthesized voice software installed so that he could communicate in the group. This had several impacts: first, the child could follow the reading, think of an idea to share and structure the idea; second, he could share the idea with the group and contribute to the gathering; third, the other children could realize that their classmate wanted and was able to communicate with the others, and even “heard his voice” for the first time; fourth: new opportunities for communication and the sharing of knowledge, experiences and thoughts appeared in the group. These changes did not occur until the teachers considered how they could improve the child’s participation in DLG, so it was the SEA that encouraged teachers to mobilize the resources that enabled the child’s participation and made these changes possible.

Importantly, the improvements achieved have been sustained and even increased through time as the implementation of SEAs also increased. Awareness of improvements has spread in their communities and that has led, in some cases, to an increased demand to enroll students with special needs in these schools, as Sandra explains in the quote below; this is another way in which the participation and learning opportunities of students with special needs in SEAs have been enhanced:

More families are coming with children with special needs that attended other schools. (…) Here, in the town, all the families know each other. (…) They talk and explain their experiences… and therefore many are requesting a change of school. (Sandra)

### Social Impact 2: Transforming Schools’ Approaches to Meeting Students’ Diversity in Terms of Special Needs

The education of students with special needs has changed not only because of increased opportunities to participate in SEAs but also because the dialogic, interactive and transformative approach behind the SEAs has been assumed by the teachers and the entire community to change the way they approach the education of these students at every moment – within and outside SEAs – now being more dialogic, interactive and transformative as well.

Before implementing SEAs, schools tended to respond to students’ special needs through individual attention, often outside the classroom and based on low expectations; they understood the students’ disabilities as an indicator of what the students could achieve. The participation of students with special needs in SEAs has meant a turning point in the schools’ approach to diversity.

#### A Focus on Interactions to Enhance Students’ Learning Opportunities

Knowing SEAs and their scientific and theoretical bases, especially the relevance of interactions for learning, has meant that teachers who have incorporated SEAs in their schools focus on the interactions they promote. In mainstream schools, the more diverse and rich interactions students found in SEAs was an element that convinced teachers to include the participation of students with special needs and to do the necessary material adaptations to allow that interaction. They could observe improvements in typically developing children, both in learning and coexistence, as a consequence of participating in SEAs, which also encouraged them to include students with special needs and extend these benefits to them, overcoming previous ideas about special education, as Irene explained:

We were intoxicated with the idea that [mainstream] students make progress, but those with special needs need different things, need that we adapt to their learning level… But we have advanced in inclusion as we have been implementing SEAs, because we realized that children with special needs can participate too, and interactions with peers are positive for them to progress, besides self-esteem, seeing they are capable, and that they can improve. (Irene)

In the context of special schools, interactions are also a topic of discussion now, which helps teachers focus on providing their students with the best learning environment possible. For the professionals working there, this has meant an opportunity to give their students richer learning opportunities within their segregated placement:

In our school program, we include what the students will learn, but we also consider and talk very much about the interactions they will have, which is a topic we had never discussed before knowing about SEAs. We focus on the type of interactions they have, if they are quality interactions, if they can have more quality, how we can promote them through SEAs, IG, DLG… (Marta)

Evidence, the SEAs, which explain what is best for our students, give us confidence in our work. We know our way to advance in giving the best results to our students. Therefore, we think of interactions; since they are segregated, we consider which type of interactions we should offer to them. (Ana)

#### Development of Scientific Thinking About Education

Another consequence of being aware of the benefits of SEAs for students with special needs, as demonstrated by research, has been the development of a more scientific way of thinking among the teaching staff. The teachers interviewed, as well as other teachers in their schools, read scientific publications emerging from or related to this line of research and discussed them in dialogic pedagogical gatherings. This helped them become familiar with research and scientific evidence, and they now look for this evidence when they must make decisions on their students’ education:

Now we say: “But is there evidence for it? Let’s see who has written about that” (…) for instance, when we are working on autism, [we want to know] if what we implement is based on scientific evidence or not, and what the most recent research about autism says. It has emerged from having implemented evidence and talking about evidence. (Marta)

Once the teachers learned that there is scientific evidence behind the success achieved by SEAs, they looked for evidence-based actions, practices or programs in any aspect of their professional activity, which increased their chances of enhancing students’ education, not only when the students participate in SEAs but at any moment they are at school. SEA participation therefore increases the potential social impact that other research in education and psychology of education can have, as these teachers look for the evidence of previous improvements achieved and reported by this research to transfer them to their own context and achieve similar improvements.

#### Changing Teachers’ Minds and Talk About Students With Special Needs

In relation to the scientific view of education, teachers have changed the way they think and talk about their students, focusing not on the students’ disabilities but on what the teachers can do to transform the educational context and improve the education of such students. These teachers do not ask whether students with special needs can participate in SEAs; they start from the premise they can, and they think on the way they can facilitate their students’ participation through, for instance, necessary adaptations. These teachers believe that this way of thinking about their students has made them improve as teachers, as their professional performance is permeated by language of possibilities:

We realize that we have a different approach, I mean, [we think about] *how* are we going to include these students or *how* are we going to promote interactions with them. And we did not have this perspective before. As a school, having had scientific evidence within reach made us improve our teaching practice, reconsider many things, and find meaning. (Marta)

At the personal level, we have improved our dialogues about what is best for our students. We are advancing in this direction, always putting the focus on the students, on what we will achieve, on the fact that this is the best for them. (Ana)

#### Rethinking and Reorganizing Specialized Support Within and Beyond SEAs

Implementing SEAs with students with special needs entailed rethinking the role of special education teachers, speech therapists and other specialized support. While these professionals used to work outside the class to provide individualized support to students with special needs, usually based on different curricular material of lower academic level, when SEAs started to be implemented, teachers agreed with these specialists that students with special needs would not leave the classroom. Instead, these professionals started to enter the classroom to support students in IG. When the class was not organized in IG, teachers kept the criteria of organizing heterogeneous groups of students to facilitate the inclusion of students with special needs, and specialists also provided support there. Speech therapists, who, in some cases, were more reluctant to change their role into a more inclusive role, also agreed to participate in SEAs by preparing activities for IG or supporting students in DLG.

One of the first things we were clear about was that these students would not leave the classroom and would be distributed within the classroom in heterogeneous groups, and at the same time, we started working in IG and DLG. (…) In my school, all of them used to leave the classroom and had different curricular materials. Objectives were set with very low expectations, low academic objectives, and then we engaged in debates and there were several changes. (…) On the one hand, the role of the speech therapist changed, and this was difficult to achieve because they felt they had lost their identity, their role, (…) but now we work and plan children’s learning together. (Sandra)

In some cases, reading and discussing research publications, such as INCLUD-ED results (INCLUD-ED Consortium, 2009), helped in organizing students and supporting them in a more inclusive way when working in SEAs and beyond, which supported the decision to maintain students with special needs in the class when SEAs were implemented:

Little by little, we saw that all students improved, and we started to do pedagogic gatherings. For instance, I remember that we discussed INCLUD-ED “Actions for success in schools in Europe,” and we emphasized the topic of groupings with the teaching staff because the special education teachers had the idea that they had to take the students with special needs out of the classroom. So, we agreed that when we worked in SEAs, these students would stay in the classroom so that they could participate in the same activities as everyone else. (Ines)

#### Higher Expectations and Enhanced Learning: Teachers Recovering Meaning in Their Profession

Being aware of SEAs and the improvements promoted and having the opportunity to discuss them and implement them in their school facilitated teachers’ enhanced belief about their students’ potential and, at the same time, gave them the tools to make that potential real; as teachers’ expectations were raised, students’ performances also raised and even surpassing these expectations. This has had an impact on students but also teachers, as some of the teachers reported rediscovering meaning in their profession as a result of being better able to facilitate the learning of students with more difficulties:

I think that the teachers who implement SEAs with our students have found more meaning in teaching, because we see that they learn. We have had high expectations, and even with these high expectations, many times, they have surprised us. We’ve said “I never imagined it could but it happened”, even if we always had high expectations. Sometimes, unintentionally, working with disabilities, we think, “well, we have high expectations, but we will get there one day”, and we are already there. (Marta)

The higher expectations and the possibilities enabled by them has meant a shift, especially in the context of special education, where low expectations and low educational levels predominate, as Ana reflected:

I think that in special schools we can easily find the “happiness curriculum”, that is to say, “poor kids, they have enough with their disability, instead of trying to learn more [let’s make them happier]”. I have worked in several special schools, and I always found colleagues with this attitude. Then, I think that implementing SEAs, and now with the line of research, I think we have realized that we have to change our minds, through dialogue: Why expect less? Let’s go for high expectations, for the best of each student, and see what we can achieve. I think it has been something that has spread in the school, as a result of starting to work in this way with students and other colleagues seeing the results. (Ana)

Importantly, the higher expectations supported by the previous evidence of improvement achieved through SEAs have made it possible for teachers to take on challenges that they would not have taken on before. For instance, Carmen explained that once she learned about the SEAs and their impact at a conference, she decided to implement these actions with the most challenging group at her school. The groups with most challenging students are often those that teachers do not choose to work with and are assigned to the least experienced teachers or those who arrived most recently at the school; however, SEAs make teachers more confident in their ability to improve these students’ educations and, as occurred in the case of Carmen, make them wish to teach precisely the most difficult groups because they know they can make a difference in the education of those students:

I could not understand how it was possible to respond to the diversity we had in the classrooms. I remember that when I arrived at the school I couldn’t, I was overwhelmed, and I remember going at the international conference and seeing it crystal clear. I saw it so clear that I remember we had a class in the school with much diversity, a very special group, and I went to the principal’s office and said, “I need to take this group and implement what I know, what the evidence says that works, to ascertain that it works, and to transform this group”. (…) And the change was amazing. (…) Now I cannot see it in any other way, because now, I feel that any challenge I face, I will succeed. And now, I feel very much like taking the group most in need, the most vulnerable one. For me, it has been awesome working like this. (Carmen)

### Social Impact 3: From Mainstream to Special Education Settings: The Transference of SEAs

The expansion of SEAs to new schools has entailed SEAs reaching new educational contexts, some of which are specific contexts in special education. Reaching these new contexts has entailed the opportunity for more inclusive, quality education.

#### SEAs as a Way to Include Students Segregated in Special Education Classes in the Mainstream Class

As the teachers reported, the inclusion of all students with special needs in SEAs has sometimes been a process, especially when the school serves students with severe disabilities, which directly affects areas of curricular learning. Irene’s school contains a specific classroom for students with language and communication disorders (a communication and language classroom) related to the autism spectrum disorder, which serves students from different municipalities. These students have little or no development of oral language, which makes it difficult for them to participate in actions such as IG and DLG, as such methods are based on dialogue and interaction. Teachers relied from the beginning on research evidence for including in SEAs other students with special needs who attended the mainstream classes. Subsequently, guided by the evidence of the improvements achieved with these students in the school, the students from the communication and language classroom also started to be included in the SEAs.

The students of mainstream classrooms started to participate first. (…) At the beginning, the students with autism, who had many difficulties, who could not speak, did not participate in SEAs; we had not thought about that yet. (…) We still had to break with the idea that we had to teach students with special needs outside the classroom. Then, when we started to include them in the classroom, especially by participating in SEAs, and we saw that the students with difficulties – but who could speak – improved, then we said, “And the other students? The most difficult ones? Let’s see if it is possible”. And it is possible. (Irene)

In this case, the teacher highlighted the importance of adapting some aspects of the development of the activity to facilitate the progressive participation of these students. In the case of students with autism and little language development, the readings for the DLG were prepared with pictograms so that the children could follow the story and express themselves. The teachers prepared the reading and the contribution for the gathering with their families ahead of time. In IG, the students started participating in only one activity, with additional support if necessary, and progressively participated in two or more activities of the IG session.

IG and DLG have made the participation of students who previously shared little learning time with their peers possible in the mainstream classroom, which means that SEAs have had an impact on their educational inclusion and learning opportunities. Furthermore, some of these students have left the communication and language classroom and are now enrolled full time in the mainstream classroom. SEAs have helped to make the possibilities for these students to learn in mainstream inclusive settings more visible and, as a consequence, some students have left open places in the communication and language classroom that can be occupied by students who are now attending special schools. Therefore, in this case, SEAs not only promote the inclusion of students within that school but also open possibilities for more inclusive trajectories for students in other schools.

Pau is a child who came to the communication and language classroom as a child with autism. Today, after having worked with him in the mainstream classroom of peers of the same age, participating in SEAs with interactions has improved his performance at the social level, and now this child is in the mainstream classroom and has left an available place in the communication and language classroom for other children with language difficulties at special schools. That is, we have achieved students who were schooled in the communication and language classroom now being in the mainstream classroom. (Ines)

#### Transference of SEAs in Special Schools

In the context of Spain, where the research was conducted, 17% of students with special needs are enrolled in special schools (World Health Organization, 2011). According to national law^5^, these are students with disabilities whose special educational needs cannot be met in mainstream schools, the most frequent types of disabilities including intellectual disabilities (36%), multiple disabilities (24%) and pervasive developmental disorder (19%)^6^. Some special schools concerned with providing the best education to their students have also wondered about the possibility of bringing to their schools the educational actions that transformed the education of students with disabilities in mainstream schools. Today, there are 4 special schools that implement SEAs. In Marta and Ana’s school, when the teaching staff started to implement SEAs, no one there had previous experience with implementing SEAs in special schools, so they had to recreate the SEAs in the new context. To ensure proper implementation to achieve the benefits that had already been observed in mainstream schools, they implemented the SEAs progressively and assessed the ongoing results:

In our school, we started with one classroom, and little by little, the number of classrooms that implemented DLG increased. Today, not only has this implementation been sustained, but the number of participants has increased, both in DLG and IG. (…) The results are very positive, because in primary education, at the beginning, only one classroom participated and now 5 entire classrooms participate. (Ana)

The transference of SEAs in their special school was not only sustained but, in time, also increased: similarly to what occurred with mainstream schools, more SEAs have been implemented in more groups and with more diverse children: “We included first children with more speech ability, and little by little we included students with less speech ability to see how we could manage to guarantee that all of them could participate in the SEA” (Marta). “Now, when you look at the timetable for the next school year, you can see that it is full of SEAs” (Ana). This extension in the implementation of SEAs in the school cannot be separated from the students’ progress: it is the improvements such work has achieved that encourages teachers to extend IG and DLG to new groups, students and learning content.

In some cases, preparing the activity with the children ahead of time facilitated their incorporation into the SEA. According to the teachers, using such strategies has made it possible for approximately 80% of the students of preprimary and primary education to participate in IG, and approximately 40% of the students to participate in DLG. In this context of special education and with this group of students, SEAs have also demonstrated improved learning. Language is an area in which most of these students present difficulties, and it is the area of improvement that the teachers have highlighted most, along with an improved coexistence between students:

With the SEAs, new language structures appeared in the students that we never imagined before that they could develop, reasoning, argumentation, first with much help and modeling, but finally, it appeared spontaneously. Then, reading books, which we did not foresee either (…). Expanding their vocabulary. (…) With the SEAs they gain richer vocabulary too. (Marta)

The main results we have seen are improvements in language competence and the quality of their contributions, sentence structures of greater complexity, improvement in explaining their opinions, improved coherence of discourse, taking into account the topic of the debate. (…) Then, an increase in the number of participants in the DLG, better knowledge of the other participants, the creation of new bonds and friendships, and the reduction of coexistence problems. (Ana)

This evidence suggests that SEAs are not limited to a particular type of school or student population but can be effective with very different types of student diversity and educational contexts. According to the teachers, the research on SEAs and special needs had an influence on these improvements achieved in their school:

I think that we could not have achieved it if we would not have this line of research and impact. I mean, it has given us much robustness, a great deal of science to say, “Okay, it has been studied, it works,” and this robustness helped us to transfer, sometimes we say “to recreate,” the SEAs. (Marta)

#### Building Collaboration Between Special and Mainstream Schools

The replicability of SEAs in special schools was accompanied by the previously mentioned transformation of teachers’ understanding of the education of students with special needs. The focus on learning interactions that both IG and DLG have led teachers in these schools not only to ensure maximum diversity in the interactions within the school by, for instance, grouping students with different disabilities and with different capabilities together, but also to look for interactions with typically developing peers who are educated in mainstream schools. Sharing learning activities with these children in an inclusive learning context entailed new learning opportunities for the special school students in a more normalized environment that could eventually help them prepare for a transition from special education to combined (special-mainstream) education. As Ana explained, in her school, the idea of collaborating with mainstream schools emerged from the high expectations developed and the will to pursue more ambitious objectives for their students. Now, the teachers want to establish this beneficial experience as a regular collaboration and extend it from DLG to IG.

Since we started to work with DLG with our students, we have seen that our objectives in DLG are changing, the same way they are progressing and improving, there is an evolution. They start to structure sentences, ask questions, talk about the topic; then, we see the need to look for higher expectations, that is, there is always a bit more. Then, we thought that as we wanted a bit more, and the ones who could provide it as role models were students of their same age. We wanted them to participate in DLG in the most normalized way possible. (…) When we did it with the [mainstream] school it was a spectacular experience, because new dialogues emerged, our students participated very much spontaneously. (…) Then, the need to create these DLG as something periodical and systematic for the next years emerged, starting with DLG, and then we will continue with IG. Each time a bit more, more inclusion, more interactions, more communication, and more learning. (Ana)

According to the teachers’ experience, the students of both regular and mainstream schools benefited from this experience. The special education students could improve their language and academic learning and find new contexts where they could be accepted and respected, and the mainstream school students had the opportunity to learn more about people with disabilities, including their difficulties and capabilities and how to interact with them, which is learning for life. In one of the mainstream schools that had less experience in implementing DLG, students could even learn from the greater experience students from the special school had with DLG. For both groups of students, many of whom lived in the same town, this collaboration entailed the possibility of coming to know each other and creating bonds beyond the school context:

For the children of the mainstream school, it has brought the opportunity to know students with disabilities and learn how to interact with them; for instance, they live in the same municipality, and maybe they met in the street and they did not interact, they did not know how to talk to each other. The gatherings are above all respect, humanity, a climate of total acceptance, that many times we do not find in society. And our students were able to demonstrate what they knew and had no problem raising their hands and sharing what they knew. Although they needed help, they asked questions to their peers at the mainstream school. I mean, their concerns, their language improvement, I think that apart from the academic and the language improvement, regarding human values it is helping both the mainstream and the special school. (Ana)

The fact that SEAs have been replicated in special schools and there are therefore mainstream and special schools that implement SEAs in the same geographical context has made these collaborations and new learning opportunities for both groups of students possible.

#### Reaching Other Educational Contexts in the Community

The research on SEAs and students with special needs has also had an impact in other places of the community beyond the school context. An illustrative example explained by Sandra is an association dedicated to people with disabilities that offers activities for children with disabilities. The fact that SEAs are open to the community facilitated the president of the association participating in IG in Sandra’s school, showing how both learning and coexistence improve in IG. In addition, the mother of a child with special needs at Sandra’s school is an active member of the association. These connections with members of this association have caused a change in the association, which is now more oriented toward promoting academic learning among the children and is more impregnated with high expectations, creating new contexts where learning and inclusion can be enhanced:

This summer, the association is promoting instrumental learning for the first time; they are doing homework, which they had done never before, and they are very satisfied. And also, the issue of the evidence (…) she told me the other day that a girl with autism had come, the family explained that she had behaviors such as pulling hair, pushing, and she told them, “Don’t worry, we are changing it”, and the mother was very happy, and she was happy too (…) They are learning to read and write since preprimary, and the families are really satisfied… This has been a big change, because they did not think like this before. (Sandra)

### Factors Supporting Social Impact

The interviews held with teachers about the impacts achieved have also shown several factors that contributed to these impacts, mainly via supporting the sustainability and replicability of the actions and the promoted improvements. It is important to summarize these factors here because taking them into account may contribute to an enhanced social impact of the research.

#### Teachers’ Permanent Training Based on Scientific Evidence

As mentioned above, a more scientific approach to education was one of the impacts achieved for teachers. This was translated into the practice of regularly reading scientific texts related to their profession, which, in turn, reinforced this scientific view. Some schools organized seminars in which the teaching staff debated these texts, and in other cases, teachers attended seminars or meetings with teachers from other schools. The texts that they read and debated included articles, books and reports resulting from this line of research and other scientific publications related to teaching and learning that could help them solve problems they encountered and improve their practice. According to the interviewed teachers, sharing this space of learning and debate has been a help in replicating the SEAs in new classes or schools and in bringing the SEAs to more students; it has also been a source of sustainability when the barriers found in the implementation of SEAs were shared and discussed:

Training is essential. As we read about the evidence and debated it, if we had a preconceived idea, we said, “No, this is true, it is as you say, this girl may be able to do that”. I think that training has been and still is essential for all this, because theory gives us a clue to put everything into practice. If we know the theory, then it is very easy. (…) We have to know first, we have to learn first. And then we see it very clearly. (Carmen)

Gaining confidence and feeling empowered to implement the actions that are supported by research has also been an effect of the teachers having access to scientific knowledge:

We emphasized very much that evidence says that children improve more with inclusion, that is, not taking them out of the classroom, if you do not group them separately… Then, you get empowered, and say, “This is really what we have to do; every time, if we could do that it would be ideal”. Then, you change your outlook a bit. (…) Because we came from another paradigm, we had another trajectory, and with the training we started to see things more clearly; we got empowered and said, “It has to be done this way, it is demonstrated that it is best, so let’s do it”. (Ines)

#### Teamwork and Networks of Support

Another facilitator of social impact highlighted by the teachers was the availability of a network of support among teachers and schools. The previously mentioned seminars are one place where some of these networks have been created. The previous experiences of other colleagues that are shared in these spaces have encouraged new teachers to implement SEAs and have also helped solve doubts and difficulties in the implementation of SEAs. These networks of support have made possible, for instance, collaborations between special and mainstream schools in sharing SEAs. Irene explained that this was an important factor in her case; the same way that special schools took the progress achieved in mainstream schools as a reference to replicate the SEAs in their context, Irene’s school took one special school as evidence of the possibilities for successfully implementing SEAs in her school:

The more positive, inclusive, rich, high expectations and interactions you have in more contexts with other professionals that are implementing SEAs in other schools and see that it is possible, that a special school is doing it and it is possible, and they improve… the more things like that you listen to throughout your professional life, and the more people you can share these spaces with, the more you empower yourself… and finally, you do it, because you believe it is true that it is possible and that you are going to make it. (Irene)

#### Recording Results and Being Aware of the Improvements Achieved

For teachers, it was also important to have a record of the students’ results related to the SEAs so that they could register progress and be fully aware of the improvements achieved. Some schools had more systematic records than others, and some of them were aware that they had to improve their recordkeeping, but all of them agreed on the importance of gathering this evidence, as it demonstrates teachers they are doing well and encourages the continuance of their work:

Results, because in daily life the inertia doesn’t let us see progress, but it is very important to talk about it with colleagues: “Look at what we have achieved,” “Look, this child could not do that and now he does”. When we verbalize it, we realize all we are achieving. (Carmen)

#### Sharing the Impact of SEAs With Families

When schools share the development and outcomes of SEAs not only among teachers but also with students’ families, the latter also become active supporters of these actions. This information can be shared in the schools’ seminars or assemblies that are open to families, in individual meetings with the family of a particular student, or while developing the SEAs if families participate, for example, as volunteers in IG. This information has led, for instance, to families not authorizing their children with special needs to receive support outside the classroom – because they know their children can progress further by participating in SEAs in their classroom—or agreeing that their child can stay in the school one more year so as to continue taking advantage of learning in SEAs.

## Discussion

Within education, I think that special education is the great forgotten area, and, with this research, I really believe that now is our time. I think that special education is starting to be visible and show that with them [students with disabilities], it is also possible. (…) I believe that it is our moment and I hope that this research helps all students and that finally, inclusion becomes a reality that we achieve between all. (Ana, teacher at a special school)

Ana, with these words, tried to synthesize what the actual and potential social impact of the line of research was for teachers such as her. Research conducted on SEAs and students with disabilities and other special educational needs allowed the identification of benefits that these educational actions entailed for these children in the schools that were already implementing them. Subsequently, this evidence has reached new schools, bringing these improvements to new student populations and improving teachers’ professional experiences, thus achieving a social impact that, as Ana said, is contributing to transforming special education.

This line of research is an example of the body of research in the psychology of education that studies several aspects of the education of students with special needs, creating interventions that improve their learning and coexistence with peers or bringing forth scientific evidence on which effective educational programs can be based. As interaction and dialogue are key components of SEAs, we argue that the evidence collected on the impact of SEAs on students with special needs shows the transformative potential of the sociocultural approach of learning (Vygotsky, 1980; Bruner, 1996) for the education of these students. Because evidence on the social impact of this line of research was obtained from a limited number of interviews, conclusions must be cautiously made. However, there is evidence supporting the achievement of social impact. The criteria defined by SIOR^7^, the Social Impact Open Repository that aims at monitoring, evaluating and improving the impact of research, enables the analysis of how social impact is approached, as well as the limitations that can be addressed to further enhance social impact achievements.

(1) *Connection of research with the social priority goals of sustainable development.* The line of research responds to *UN Sustainable Development Goal 4 on Quality Education:* Ensure inclusive and equitable quality education and promote lifelong learning opportunities for all. Therefore, the research is aligned with one of the social priorities.
(2) *Percentage of improvement achieved regarding the departure situation.* The interviews conducted allowed the collection of evidence on the improvements achieved in terms of the students’ learning and improved coexistence and the schools’ more efficient response to student diversity. However, an accurate and quantified record of the academic and/or social improvements of these students has not been systematized. Therefore, evidence of the social impact would be enhanced with a more systematic procedure to collect and quantify the improvements.
(3) *Transferability of the impact: the actions based on the project’s results have been successfully applied in more than one context.* Transferability has been achieved in different directions: first, replicating the SEAs in mainstream schools with the participation of students with special needs in these schools; second, recreating the SEAs in special schools, thus transferring the actions to a new student population; and third, transferring the SEAs to other out-of-school educational contexts in the community.
(4) *Scientific, political, and social dissemination.* The benefits of SEAs for students with disabilities and other special needs have been disseminated through scientific publications, conferences and training for teachers and schools. Importantly, this dissemination has been a key component for the transferability and sustainability of the impact, according to the evidence collected and is associated with the scientific training of teachers, who used such publications to learn from and discuss the evidence and transform their own professional practice.
(5) *Sustainability of the impact achieved.* According to the evidence collected, in all the new contexts and new populations of students where SEAs have been transferred, the intensity of the implementation has not only been sustained but also increased, and the same occurred with the improvements achieved. Although an accurate quantification of the improvement is not yet available, the experience of the sample of teachers who were involved in the transference of the SEAs and still implement them in their own context points in this direction.

Taking this into account, further research on SEAs and students with special needs with social impact could cover four aspects. First, to analyze how SEAs put into practice contributions from theory in the psychology of education to support the learning and development of children with special needs more in depth. Second, to define a procedure to collect and quantify the improvements achieved by the students as a result of participating in SEAs. The INTER-ACT project, which is currently advancing this line of research, will contribute to quantifying this improvement and strengthening the evidence of the research’s social impact. Third, to support the transference of the SEAs and the improvements associated with them to new schools. Additional impact is foreseen in this regard, as the ongoing INTER-ACT project will transfer SEAs to new mainstream and special schools and will add further evidence on the key elements for the transferability of SEAs to new contexts with students with special needs and those without. Finally, to extend the interactive understanding of learning and development beyond schools and the teaching and learning contexts, reaching other related professionals and activities, such as evaluation, attention and counseling related to special needs; these areas of intervention are still very much impregnated with an individualistic perspective more aligned with the medical model than with the social model of disability, and students and schools would benefit from coordinated work based on the evidence of the benefits of the interactive approach of SEAs.

## Data Availability Statement

The datasets generated for this study are available on request to the corresponding author.

## Ethics Statement

The study was fully approved by the Ethics Board of the Community of Researchers on Excellence for All (CREA). The participants provided their written informed consent to participate in this study.

## Author Contributions

SM conceived the original idea with the support of ER. ED conducted the literature review. SM with the support of RG analyzed the results of the line of research (case studies) from the perspective of social impact. ER coordinated the data collection (interviews) on social impact. SM conducted the interviews, and transcribed and analyzed them with the support of RG and ED. SM wrote a full draft of the manuscript. ED, ER, and RG revised it and included corrections. SM revised the final version of the manuscript.

## Conflict of Interest

The authors declare that the research was conducted in the absence of any commercial or financial relationships that could be construed as a potential conflict of interest.

## Footnotes

1 The Ethics Board was composed of Dr. Marta Soler (president), who has expertise in the evaluation of projects from the European Framework Programme of Research of the European Union and of European projects in the area of ethics; Dr. Teresa Sordé, who has expertise in the evaluation of projects from the European Framework Programme of Research and is researcher in the area of Roma studies; Dr. Patricia Melgar, a founding member of the Catalan Platform Against Gender Violence and a researcher in the area of gender and gender violence; Dr. Sandra Racionero, a former secretary and member of the Ethics Board at Loyola University Andalusia (2016-2018) and a review panel member for COST action proposals in the area of health; Dr. Cristina Pulido, an expert in data protection policies and child protection in research and communication and a researcher in communication studies; Dr. Oriol Rios, a founding member of the “Men in Dialogue” association, a researcher in the area of masculinities, and the editor of “Masculinities and Social Change,” a journal indexed in WoS and Scopus; and Dr. Esther Oliver, who has expertise in the evaluation of projects from the European Framework Programme of Research and is a researcher in the area of gender violence.

2 Schools as Learning Communities in Spain: http://utopiadream.info/ca/centros-en-funcionamiento/caracteristicas/

3 Schools as Learning Communities in Europe: Successful Educational Actions for all (SEAS4ALL). ERASMUS + Programme. Record number: 2015-1-ES01-KA201-016327. https://seas4all.eu/; Social transformation through Educational Policies based on Successful Educational Actions (STEP4SEAS). ERASMUS + Programme. Record number: 11. 580432-EPP-1-2016-1-ES-EPPKA3-IPI-SOC-IN. https://www.step4seas.eu/

4 2018 Report of the Schools as Learning Communities network in Latin America https://www.comunidaddeaprendizaje.com.es/uploads/materials/579/352de6fce741a0d1e6d17c67944cec2c.pdf

5 Ley Orgánica de Educación [Organic Law of Education] (LOE), of 2006, amended by the Ley Orgánica para la Mejora de la Calidad Educativa [Organic Law for the Improvement of Quality of Education] (LOMCE), of 2013.

6 Spanish Ministry of Education and Vocational Training. Statistics on Education. School year 2017-2018. https://www.educacionyfp.gob.es/servicios-al-ciudadano/estadisticas/no-universitaria/alumnado/matriculado/2017-2018-rd.html

7 https://sior.ub.edu/indicators

## References

[B1] Akhutina T. Foreman N. Krichevets A. Matikka L. Narhi V. Pylaeva N. (2003). Improving spatial functioning in children with cerebral palsy using computerized and traditional game tasks. *Disabil. Rehabil.* 25 1361–1371. 10.1080/09638280310001616358 14660204

[B2] Alanazi M. S. (2017). Use of flashcards in dealing with reading and writing difficulties in SEN students. *Multidiscip. J. Educ. Res.* 7 53–87. 10.17583/remie.2017.2211

[B3] Alenizi M. A. (2019). Effectiveness of a program based on a multi-sensory strategy in developing visual perception of primary school learners with learning disabilities: a contextual study of Arabic learners. *Int. J. Educ. Psychol.* 8 72–104. 10.17583/ijep.2019.3346

[B4] Anderson D. L. Watt S. E. Shanley D. C. (2014). A multi-dimensional model of the origins of attitude certainty: teachers’ attitudes toward attention-deficit/hyperactivity disorder. *Soc. Psychol. Educ.* 17 19–50. 10.1007/s11218-013-9235-5

[B5] Asmus J. M. Carter E. W. Moss C. K. Biggs E. E. Bolt D. M. Born T. L. (2017). Efficacy and social validity of peer network interventions for high school students with severe disabilities. *Am. J. Intellect. Dev. Disabil.* 122 118–137. 10.1352/1944-7558-122.2.118 28257242

[B6] Auphan P. Ecalle J. Magnan A. (2019). Computer-based assessment of reading ability and subtypes of readers with reading comprehension difficulties: a study in French children from G2 to G9. *Eur. J. Psychol. Educ.* 34 641–643. 10.1007/s10212-018-0396-7

[B7] Bowles D. Radford J. Bakopoulou I. (2018). Scaffolding as a key role for teaching assistants: perceptions of their pedagogical strategies. *Br. J. Educ. Psychol.* 88 499–512. 10.1111/bjep.12197 29152714

[B8] Bruner J. S. (1973). *Beyond the Information Given: Studies in the Psychology of Knowing.* New York, NY: WW Norton.

[B9] Bruner J. S. (1996). *The Culture of Education.* Cambridge, MA: Harvard University Press.

[B10] Christou M. Molina S. (2009). Educational inclusion and critical pedagogy. *Teor. Educ.* 10 31–55.

[B11] Chun Sik Min (2010). Cultural discourse on special education’s identity. *J. Intellect. Disabil.* 12 213–229.

[B12] Dainez D. Smolka A. L. (2014). The concept of compensation in the dialogue of Vygotsky with Adler: human development, education and disability. *Educ. Pesqui.* 40 1093–1108. 10.1590/S1517-97022014071545

[B13] Elboj C. Niemelä R. (2010). Sub-communities of Mutual Learners in the Classroom: The case of Interactive groups. *Rev. Psicodidáctica* 15 177–189.

[B14] European Agency for Special Needs and Inclusive Education (2017). *Early School Leaving and Learners with Disabilities and/or Special Educational Needs: Final Summary.* Available online at: https://www.european-agency.org/resources/publications/early-school-leaving-and-learners-disabilities-andor-special-educational-1 (accessed March 3, 2020).

[B15] European Commission (2010). *European Disability Strategy 2010-2020: A Renewed Commitment to a Barrier-Free Europe. Brussels: European Commission.* Available online at: https://ec.europa.eu/eip/ageing/standards/general/general-documents/european-disability-strategy-2010-2020_en (accessed March 3, 2020).

[B16] European Commission (2013). *Ethics for Researchers. Facilitating Research Excellence in FP7.* Available online at: http://ec.europa.eu/research/participants/data/ref/fp7/89888/ethics-for-researchers_en.pdf (accessed March 3, 2020).

[B17] Flecha R. (2015). *Successful Educational Action for Inclusion and Social Cohesion in Europe.* Berlin: Springer 10.1007/978-3-319-11176-6

[B18] Flecha R. Soler-Gallart M. Sordé T. (2015). Social impact: Europe must fund social sciences. *Nature* 528:193. 10.1038/528193d 26659175

[B19] Flecha R. (2006-2011). *INCLUD-ED. Strategies for inclusion and social cohesion in Europe from Education. 6th Framework Programme.* Brussels: European Commission.

[B20] Garcia R. Mircea T. Duque E. (2010). Socio-cultural transformation and the promotion of learning. *Rev. Psicodidáctica* 15 207–222. 17399849

[B21] García-Carrión R. Molina Roldán S. Grande López L. A. Buslón Valdez N. (2016). Análisis de las interacciones entre alumnado y diversas personas adultas en actuaciones educativas de éxito: hacia la inclusión de todos y todas. *Rev. Latinoam. Educ. Incl.* 10 115–132. 10.4067/S0718-73782016000100007

[B22] Garcia-Carrion R. (2018-2020). *INTER-ACT. Interactive Learning Environments for the Inclusion of Students with and Without Disabilities: Improving Learning, Development and Relationships. R+D Plan.* Madrid: Spanish Ministry of Science.

[B23] García-Carrión R. Molina Roldán S. Roca E. (2018). Interactive Learning Environments for the Educational Improvement of Students with Disabilities in Special Schools. *Front. Psychol.* 9:1744. 10.3389/fpsyg.2018.01744 30283388 PMC6157546

[B24] Gómez A. Padrós M. Ríos O. Mara L. C. Pukepuke T. (2019). Reaching social impact through communicative methodology. Researching with rather than on vulnerable populations: the Roma case. *Front. Educ.* 4:9 10.3389/feduc.2019.00009

[B25] Gómez A. Puigvert L. Flecha R. (2011). Critical communicative methodology: informing real social transformation through research. *Qual. Inq.* 17 235–245. 10.1177/1077800410397802

[B26] Gómez A. Siles G. Tejedor M. (2012). Contributing to social transformation through Communicative Research Methodology. *Qual. Res. Educ.* 1 36–57. 10.4471/qre.2012.02

[B27] Goodley D. (2001). ‘Learning Difficulties’, the social model of disability and impairment: challenging epistemologies. *Disabil. Soc.* 16 207–231. 10.1080/09687590120035816

[B28] Haegele J. A. Hodge S. (2016). Disability discourse: overview and critiques of the medical and social models. *Quest* 68 193–206. 10.1080/00336297.2016.1143849

[B29] Hatcher P. J. Goetz K. Snowling M. J. Hulme C. Gibbs S. Smith G. (2006). Evidence for the effectiveness of the Early Literacy Support programme. *Br. J. Educ. Psychol.* 76 351–367. 16719968 10.1348/000709905X39170

[B30] Heidrich R. Bassani P. (2012). Inclusive design - assistive technology for people with cerebral palsy. *Work* 41 4762–4766. 10.3233/WOR-2012-0028-4762 22317454

[B31] Holliman A. J. Hurry J. (2013). The effects of Reading Recovery on children’s literacy progress and special educational needs status: a three-year follow-up study. *Educ. Psychol.* 33 719–733. 10.1080/01443410.2013.785048

[B32] Hughes C. Foley S. White N. Devine R. T. (2018). School readiness in children with special educational needs and disabilities: psychometric findings from a new screening tool, the Brief Early Skills, and Support Index. *Br. J. Educ. Psychol.* 88 606–627. 10.1111/bjep.12206 29266202

[B33] INCLUD-ED Consortium (2009). *Actions for Success in Schools in Europe.* Brussels: European Commission.

[B34] Krull J. Wilbert J. Hennemann T. (2018). Does social exclusion by classmates lead to behaviour problems and learning difficulties or vice versa? A cross-lagged panel analysis. *Eur. J. Spec. Needs Educ.* 33 235–253. 10.1080/08856257.2018.1424780

[B35] Law Y. Lam S. Law W. Tam Z. W. Y. (2017). Enhancing peer acceptance of children with learning difficulties: classroom goal orientation and effects of a storytelling programme with drama techniques. *Educ. Psychol.* 37 537–549. 10.1080/01443410.2016.1214685

[B36] Lindsay G. (2007). Educational psychology and the effectiveness of inclusive education/mainstreaming. *Br. J. Educ. Psychol.* 77 1–24. 10.1348/000709906X156881 17411485

[B37] Lloyd J. E. V. Irwin J. G. Hertzman C. (2009). Kindergarten school readiness and fourth-grade literacy and numeracy outcomes of children with special needs: a population-based study. *Educ. Psychol.* 29 583–602. 10.1080/01443410903165391

[B38] Lopez de Aguileta G. (2019). Developing school-relevant language and literacy skills through dialogic literary gatherings. *Int. J. Educ. Psychol.* 8 51–71. 10.17583/ijep.2019.4028

[B39] Lovett M. W. Frijters J. C. Wolf M. Steinbach K. A. Sevcik R. A. Morris R. D. (2017). Early intervention for children at risk for reading disabilities: the impact of grade at intervention and individual differences on intervention outcomes. *J. Educ. Psychol.* 109 889–914. 10.1037/edu0000181 PMC9164258 35664550

[B40] Molina S. Ríos O. (2010). Including students with disabilities in learning communities. *Psychol. Soc. Educ.* 2 1–9.

[B41] Molina S. (2003-2007). *Interactive Groups: A Practice of the Learning Communities for the Inclusion of Students with Disabilities.* Ph.D. thesis, Universitat de Barcelona, Barcelona.

[B42] Montague M. Krawec J. Enders C. Dietz S. (2014). The effects of cognitive strategy instruction on math problem solving of middle-school students of varying ability. *J. Educ. Psychol.* 106 469–481. 10.1037/a0035176

[B43] Oldfield J. Humphrey N. Hebron J. (2017). Risk factors in the development of behaviour difficulties among students with special educational needs and disabilities: a multilevel analysis. *Br. J. Educ. Psychol.* 87 146–169. 10.1111/bjep.12141 28168692

[B44] Papadopoulos T. C. Charalambous A. Kanari A. Loizou M. (2004). Kindergarten cognitive intervention for reading difficulties: the PREP remediation in Greek. *Eur. J. Psychol. Educ.* 19 79–105. 10.1007/BF03173238

[B45] Park N. (2015). A study on disabilities human rights education in social studies from the viewpoint of disability studies. *J. Hum. Rights Law Relat. Educ.* 8 1–19.

[B46] Pitchford N. J. Kamchedzera E. Hubber P. J. Chigeda A. L. (2018). Interactive apps promote learning of basic mathematics in children with special educational needs and disabilities. *Front. Psychol.* 9:262. 10.3389/fpsyg.2018.00262 29559940 PMC5845689

[B47] Puigvert L. Christou M. Holford J. (2012). Critical communicative methodology: including vulnerable voices in research through dialogue. *Camb. J. Educ.* 42 513–526. 10.1080/0305764X.2012.733341

[B48] Roberts G. J. Cho E. Garwood J. D. Goble G. H. Robertson T. Hodges A. (2019). Reading interventions for Students with reading and behavioral difficulties: a meta-analysis and evaluation of co-occurring difficulties. *Educ. Psychol. Rev.* 32 17–47. 10.1007/s10648-019-09485-1

[B49] Schwab S. Huber C. Gebhardt M. Goble G. H. Robertson T. Hodge A. (2016). Social acceptance of students with Down syndrome and students without disability. *Educ. Psychol.* 36 1501–1515. 10.1080/01443410.2015.1059924 23055287

[B50] Soler M. (2015). Biographies of “Invisible” people who transform their lives and enhance social transformations through dialogic gatherings. *Qual. Inq.* 21 839–842. 10.1177/1077800415614032

[B51] Stake R. E. (2006). *Multiple Case Study Analysis.* New York, NY: Guilford.

[B52] Tzuriel D. Shamir A. (2007). The effects of peer mediation with young children (PMYC) on chldren’s cognitivie modificability. *Br. J. Educ. Psychol.* 77 143–165. 10.1348/000709905x84279 17411492

[B53] UNESCO (2017). *School Violence and Bullying. Global Status Report.* Available online at: https://unesdoc.unesco.org/ark:/48223/pf0000246970 (accessed March 3, 2020).

[B54] Valls R. Kyriakides L. (2013). The power of Interactive Groups: how diversity of adults volunteering in classroom groups can promote inclusion and success for children of vulnerable minority ethnic populations. *Camb. J. Educ.* 43 17–33. 10.1080/0305764X.2012.749213

[B55] Valls R. (2008-2011). *MIXTRIN. Ways of Grouping Students Together and How this is Related to Success at School: Mixture, Streaming and Inclusion. R+D Plan.* Madrid: Spanish Ministry of Science.

[B56] Velázquez Callado C. (2012). Analysis of the effects of the implementation of cooperative learning in physical education. *Qual. Res. Educ.* 1 80–105. 10.4471/qre.2012.04 26286447

[B57] Vellutino F. R. Scanlon D. M. (2002). The Interactive Strategies approach to reading intervention. *Contemp. Educ. Psychol.* 27 573–635. 10.1016/S0361-476X(02)00002-4

[B58] Vuorinen K. Erikivi A. Uusitalo-Malmivaara L. (2019). A character strength intervention in 11 inclusive Finnish classrooms to promote social participation of students with special educational needs. *J. Res. Spec. Educ. Needs* 19 45–57. 10.1111/1471-3802.12423

[B59] Vygotsky L. (2018). Compensatory processes in the development of the retarded child. *Educ. Pesqui.* 44:e44003001 10.1590/S1678-4634201844003001

[B60] Vygotsky L. S. (1980). *Mind in Society: The Development of Higher Psychological Processes.* Cambridge, MA: Harvard University Press.

[B61] Wilson C. Woolfson L. M. Durkin K. Elliott M. A. (2016). The impact of social cognitive and personality factors on teachers’ reported inclusive behaviour. *Br. J. Educ. Psychol.* 86 461–480. 10.1111/bjep.12118 27145063

[B62] World Health Organization (2011). *World Report on Disability.* Geneva: World Health Organization.

